# Supplementary material for: MetaRibo-Seq measures translation in microbiomes
Source: Nat Commun. 2020 Jun 29;11:3268. doi: 10.1038/s41467-020-17081-z (PMC7324362; doi:10.1038/s41467-020-17081-z)
Supplement: Supplementary file 10 — Supplementary Data 7 [file 41467_2020_17081_MOESM10_ESM.zip › File2/Confidence_VeryHigh_Taxonomy/151691_out.krona.html]

Javascript must be enabled to view this page.

members
magnitude
magnitudeUnassigned
count
unassigned
taxon
rank

151691\_out

10

superkingdom
2
10

phylum
1239
10

10
186801
class

10
order
186802

1
family
186803


SRS021153\_contig\_number\_129
species
1520827
1

186804
family
1

1520829
species
1

SRS146888\_contig\_number\_4113


SRS014287\_contig\_number\_984SRS015217\_contig\_number\_contig-100\_4522.52995SRS016495\_contig\_number\_5598SRS043768\_contig\_number\_contig-100\_10326.144249SRS053214\_contig\_number\_3846SRS143876\_contig\_number\_32476SRS147022\_contig\_number\_contig-100\_17903.17903SRS148511\_contig\_number\_contig-100\_21822.69586
species
1898207
8
